# Neutrophil Gelatinase-Associated Lipocalin as Potential Predictive Biomarker of Melanoma and Non-Melanoma Skin Cancers in Psoriatic Patients: A Pilot Study

**DOI:** 10.3390/ijms232012291

**Published:** 2022-10-14

**Authors:** Alice Verdelli, Marzia Caproni, Alessio Coi, Alberto Corrà, Donatella Degl’Innocenti, Marzia Vasarri, Lavinia Quintarelli, Valter Volpi, Emanuele Maria Cipollini, Emanuela Barletta

**Affiliations:** 1 Department of Health Sciences, Section of Dermatology, Rare Dermatological Unit, Azienda USL Toscana Centro, University of Florence, 50125 Florence, Italy; 2 Unit of Epidemiology of Rare Diseases and Congenital Anomalies, Institute of Clinical Physiology, National Research Council, 56127 Pisa, Italy; 3 Department of Health Sciences, Section of Dermatology, University of Florence, 50125 Florence, Italy; 4 Department of Experimental and Clinical Biomedical Sciences, University of Florence, Viale Morgagni 50, 50134 Florence, Italy

**Keywords:** human neutrophil gelatinase-associated lipocalin, lipocalin, psoriasis, skin cancers, non-melanoma skin cancers, melanoma, biomarkers

## Abstract

Background: Studies have demonstrated a higher risk of nonmelanoma skin cancers (NMSC) and a modestly increased melanoma risk in patients with psoriasis. To date, no biomarkers predictive of evolution have been identified yet. Methods: The aim of this prospective case-control study was to investigate the potential role of neutrophil gelatinase-associated lipocalin (NGAL) as a predictive biomarker of skin cancers in psoriatic patients. Patients with a diagnosis of psoriasis were enrolled, as well as healthy subjects and patients with skin cancers as controls. Plasma protein expression of NGAL, metalloproteinases (MMP)-2, and MMP-9 was performed by an enzyme-linked immunosorbent assay (ELISA). In all the patients who developed skin cancer at follow-up, NGAL, MMP-2, and MMP-9 serum levels were dosed again. Results: Plasma NGAL levels were significantly higher in psoriatic patients with NMSC than without (182.3 ± 36.6 ng/mL vs. 139.9 ± 39.3 ng/mL) (*p* < 0.001). Plasma NGAL levels were significantly higher (*p* < 0.00001) in patients with psoriasis and NMSC than in patients with skin tumors without psoriasis (182.3 vs. 122.9). Patients with psoriasis who developed NMSC at follow-up showed increased plasma MMP-9 levels. Conclusion: NGAL seems to play a role in the pathogenesis of NMSC but not melanoma in patients with psoriasis.

## 1. Introduction

Human neutrophil gelatinase-associated lipocalin (NGAL) is a 25 kDa secreted protein belonging to the lipocalin superfamily, which comprises small, secreted proteins with a highly conserved core tertiary structure capable of binding small hydrophobic molecules [1]. It has progressively emerged as a pleiotropic molecule involved in a variety of physiological and pathophysiological processes [2]. Initially described as a factor of the innate immune system, contributing to antibacterial iron-depletion strategy [3,4,5], in recent years NGAL has become increasingly relevant as a potential clinical biomarker in inflammatory diseases. As an acute phase protein, it is an excellent predictor of acute kidney injury, a marker of chronic kidney disease injury [6,7,8], and a predictor of unfavorable outcomes in patients with acute myocardial infarction [9,10]. It has been associated with metabolic disorders [11], and its levels are increased in patients with active inflammatory bowel diseases [12]. It has been proposed as a biomarker of cartilage degradation in arthritic diseases [13,14]. More recently, upregulation of NGAL has been reported in systemic lupus erythematosus [15] and in multiple neurodegenerative conditions [16,17]. Furthermore, NGAL levels are elevated in many human epithelial cancers [18,19] and a few studies highlighted an association between NGAL levels and high-grade malignancy, relapse proneness, metastasis, and poor prognosis [20,21]. This may be due to the fact that NGAL forms a complex with both matrix metalloproteinases, (MMP)-2 and MMP-9, in cancer cells, which are suggested to be involved in regulating cell differentiation [22,23].

Recently, some studies have demonstrated an increased expression of NGAL in calcium-induced keratinocyte differentiation, suggesting that it may have a role in the maintenance of skin homeostasis [24,25,26]. In fact, it seems to be upregulated in inflammatory skin diseases, including psoriasis [24,27,28]. Patients with psoriasis and psoriatic arthritis (PsA) have been reported to show higher serum and lesional NGAL levels than healthy subjects [29,30,31,32,33,34]. Although the involvement of NGAL in the pathogenetic mechanisms of psoriasis has not been fully elucidated yet [35], it may modulate neutrophil function [30] as it induces neutrophil infiltration, migration, and activation, and it is capable of augmenting T-helper 17 response [24,35]. Moreover, studies have demonstrated that interleukin (IL)-17 signaling pathways, either alone or in conjunction with tumor necrosis factor (TNF)-*α*, can induce NGAL expression and secretion [35,36,37]. Therefore, according to some authors, NGAL could be used as a potential biomarker for psoriasis [38,39].

In a recent study, NGAL was found to be highly increased in actinic keratosis (AK) and squamous cell carcinoma (SCC) [25]. Since NGAL has a key role in keratinocyte differentiation, its upregulation may be one trigger for tumor induction [2]. A study on melanoma showed a substantial downregulation of NGAL only in metastatic melanoma while no involvement in the primary tumor was demonstrated [40].

In this prospective case-control study, we aimed to investigate the role of NGAL in psoriasis and skin cancers. As a few studies have demonstrated a higher risk of nonmelanoma skin cancers (NMSC) and melanoma in patients with psoriasis but no biomarkers predictive of evolution have been identified yet, our main objective was to evaluate if NGAL could be considered a biomarker for the development of skin cancers in psoriatic patients. Moreover, since MMP-2 and MMP-9 are overexpressed in keratinocytes and in the serum of psoriatic patients and play a role in the pathogenesis of cancers, we aimed to investigate differences in their plasma levels in patients with isolated psoriasis compared with patients with psoriasis and skin cancers and to assess their association with NGAL. Finally, we dosed C-reactive protein (CRP), erythrocyte sedimentation rate (ESR), and 25(OH)-vitamin D levels in patients with isolated psoriasis to compare them with patients with psoriasis and skin cancers and to investigate their associations with NGAL.

## 2. Results

Demographics and clinical data are summarized in Table 1.

### 2.1. Patients with Psoriasis and Controls: Demographic and Clinical Data

A total of 98 patients affected by psoriasis (59 males and 39 females), 15 patients with skin cancers (6 males and 9 females), and 20 healthy subjects (8 males and 12 females) were included. The total mean age at baseline in all psoriatic patients was 53.8 ± 18.2 (range, 19–93) vs. 56.5 ± 10.6 years (range, 7–74) for healthy subjects. No significant differences in sex or mean age between psoriatic patients and controls were reported.

The relative prevalences of the different subtypes of psoriasis were the following: plaque-type 71.4% (n = 70); guttate 8.2% (n = 8); palmoplantar 5.1% (n = 5); inverse psoriasis 5.1% (n = 5). Ten out of ninety-eight patients presented simultaneously with two or more of these subtypes. The duration of the disease ranged from 1 to 49 years, with a mean of 17.5 ± 11.1 years. Basal PASI scores ranged from 2 to 17, with a median score of 8.5. Of the 98 patients studied, 72 (73.5%) had a mild form of psoriasis (PASI < 10) while 26 (26.5%) had the moderate (PASI 10–20) form.

Previous treatments were declared by 87.7% (n = 86) of the patients, including topical corticosteroids (55.8%, n = 48), phototherapy (16.3%, n = 14), and oral immunosuppressants (7.0%, n = 6). Eighteen patients (20.9%) underwent both topical corticosteroids and phototherapy.

The median follow-up in all psoriasis patients was 17.6 ± 7.2 months (range 4–30).

### 2.2. Patients with Psoriasis and Healthy Controls: Laboratoristic Data

Results are reported in Table 2. ELISA measurements showed that plasma NGAL concentrations were significantly higher in patients with psoriasis (144.2 ± 41.8 ng/mL) than in healthy controls (17.1 ± 11.7 ng/mL)(*p* < 0.0001). Even after adjusting for sex and age, psoriasis resulted to be strongly associated with higher NGAL levels. Plasma MMP-9 levels were also significantly higher in psoriatic patients than controls (329.5 ± 156.7 ng/mL vs. 72.4 ± 27.6 ng/mL) (*p* < 0.0001). Even after adjusting for sex and age, psoriasis resulted to be strongly associated with higher MMP-9 levels. Differently, plasma MMP-2 levels were significantly higher in controls than in psoriatic patients (170.9 ± 136.7 ng/mL vs. 58.1 ± 44.5 ng/mL) (*p* < 0.0001). Even after adjusting for sex and age, psoriasis resulted to be strongly associated with lower MMP-2 levels.

C-reactive protein (CRP) levels in patients with psoriasis were significantly higher than controls (0.4 ± 0.4 mg/dL vs. 0.2 ± 0.2 mg/dL)*(p* = *0.02).* Moreover, erythrocyte sedimentation rate (ESR) levels were also higher in patients with psoriasis than in controls (15.2 ± 9.5 mm/h vs. 8.8 ± 3.0 mm/h) (*p* = 0.004). Finally, no significant differences were found in 25(OH)-vitamin D levels between the two groups (26.1 ± 7.4 ng/mL vs. 28.6 ± 7.4 ng/mL).

In patients with psoriasis, no correlation between plasma NGAL, MMP-2, and MMP-9 levels and PASI, inflammatory indexes, or 25(OH)-vitamin D levels was revealed.

### 2.3. Patients with Psoriasis and Skin Cancers: Clinical and Histological Features

Among psoriatic patients, 15 presented a NMSC at baseline (6 females and 9 males) and 8 had melanoma (3 females and 5 males)(Table 1). Clinical and histological characteristics are reported in Table 3 and Table 4.

### 2.4. Patients with Psoriasis without Skin Cancers vs. Patients with Psoriasis and NMSC

As shown in Table 5, the mean age of patients with psoriasis and NMSC was higher than in patients with psoriasis without NMSC (71.1 ± 12.7 vs. 49.5 ± 17.6) (*p* < 0.0001).

Plasma NGAL levels were significantly higher in psoriatic patients with NMSC than without (182.3 ± 36.6 ng/mL vs. 139.9 ± 39.3 ng/mL)(*p* < 0.001). Even after adjusting for sex and age, patients with psoriasis and NMSC were found to be strongly associated with higher NGAL levels.

CRP levels were higher in psoriatic patients with NMSC than without (0.6 ± 0.6 mg/dL vs. 0.4 ± 0.4 mg/dL)(*p* = 0.04).

Comparing the mean MMP-2 and MMP-9 levels of subjects with psoriasis and NMSC with those without skin cancers, no statistically significant differences were found. Moreover, no statistically significant differences in 25(OH)-vitamin D and ESR levels were found in the two groups.

### 2.5. Patients with Psoriasis without Skin Cancers vs. Patients with Psoriasis and Melanoma

Data are reported in Table 6. Patients with psoriasis and melanoma were homogeneously distributed between males and females. The mean age of patients with psoriasis and melanoma was higher than that of patients with psoriasis without melanoma (61.9 ± 9.7 vs. 49.5 ± 17.6). However, the difference was on the verge of statistical significance (*p* = 0.05).

Plasma NGAL levels were lower in psoriatic patients with melanoma than without (112.9 ± 26.6 ng/mL vs. 139.9 ± 39.3 ng/mL). Although the difference was on the verge of statistical significance (*p* = 0.06), after adjusting for sex and age, it became significant (*p* = 0.03).

Concerning plasma MMP-2 and MMP-9, CRP, ESR, and 25(OH)-vitamin D levels, no statistically significant differences between psoriatic patients with and without melanoma were found.

### 2.6. Patients with Psoriasis and Skin Cancers at Follow-Up

The median follow-up in all psoriatic patients was 17.6 ± 7.2 months (range 4–30). In the follow-up period, three patients developed NMSC and experienced an increase in MMP-9 levels at the time of neoplasm removal compared to basal. Data are reported in Table 7.

### 2.7. Patients with Skin Cancers without Psoriasis vs. Patients with Skin Cancers and Psoriasis

Plasma NGAL levels were significantly higher (*p* < 0.00001) in patients with skin tumors than those of healthy controls (122.9 ng/mL vs. 17.1 ng/mL), even after adjustment for sex and age. In total, the mean of NGAL in patients with psoriasis and skin cancers was significantly higher (*p* = 0.013) than in patients with skin tumors only (both NMSC and M)(158.1 ng/mL vs. 122.9 ng/mL). However, plasmatic NGAL levels were significantly higher (*p* < 0.00001) in patients with psoriasis and NMSC than in patients with skin tumors without psoriasis (182.3 ng/mL vs. 122.9 ng/mL), even after adjustment for sex and age.

## 3. Material and Methods

The study was approved by the local Ethic Committee (cod. 12655_bio).

### 3.1. Patients’ Enrolment

Patients with a clinical and/or histological diagnosis of psoriasis, regardless of the disease onset, were enrolled at the Department of Dermatology at the University of Florence from January 2017 to January 2020. The exclusion criteria included: age < 18 years; history of PsA; history of malignancy, other than skin cancers; systemic diseases, including cardiovascular, nephrological, endocrinological, and gastrointestinal diseases; acute/chronic infection; topical/systemic treatment for psoriasis in the previous 6 months; pregnancy or up to 6 months postpartum period. As controls, enrolled healthy subjects and patients with skin cancers underwent annual total body dermoscopic examinations.

Before starting the study, written informed consent was obtained from all the participants. After enrollment, a standardized data collection form was used for each patient and included: demographic data, Fitzpatrick’s skin type, medical history, comorbidities, family history of psoriasis and/or skin cancers, age at diagnosis, clinical features (type and site of psoriasis), the severity of psoriasis according to the *psoriasis area and severity index score* (PASI), and duration of psoriasis.

At baseline, defined as the first visit (t0), a clinical and dermatoscopic evaluation were performed on each enrolled patient and then repeated every 4 months during the study period. Suspected skin cancers (melanoma and/or NMSC) were surgically removed and a histological examination was carried out.

### 3.2. Plasma Collection

After enrollment, three blood samples were collected from each patient with psoriasis and/or with skin cancers as well as from controls. All blood samples were collected after obtaining written informed consent from the donors.

#### 3.2.1. NGAL, MMP-2, and MMP-9 Detection with Enzyme-Linked Immunosorbent Assay (ELISA)

One peripheral anticoagulated blood sample was obtained from the study and control group using EDTA-containing vacutainer tubes and then centrifuged for 15 min at 1000× g. Plasma was collected and stored at −70 °C until used. Levels of plasma NGAL, MMP-2, and MMP-9 were detected by ELISA, using a commercial kit (NGAL human ELISA Kit, Bioporto Diagnostic, Hellerup, Denmark; MMP-2 and MMP-9 human ELISA Kit, ThermoFisher Scientific Inc., Waltham, MA USA). A blood sample was collected from all the patients who developed skin cancer at follow-up. In this subgroup of patients, NGAL, MMP-2, and MMP-9 plasma levels were re-dosed each time a new skin tumor was surgically removed. Values were reported in ng/mL.

#### 3.2.2. 25(OH)-Vitamin D Dosage

25(OH)-vitamin D plasma level was assessed in each enrolled patient and controls by direct competitive chemiluminescent immunoassay (Liaison© 25-OH Vitamin D Total Assay, DiaSorin S.p.A.,Saluggia, Italy). Normal 25(OH)-vitamin D plasma levels were defined as follows: normal ≥ 30 ng/mL; insufficiency = 10–30 ng/mL; severe deficiency < 10 ng/mL.

#### 3.2.3. Detection of CRP and ESR

CRP was dosed using a commercially available turbidimetric method (CRP Ultra, Sentinel Diagnostic S.p.A.,Milano, Italy)(detection limit > 0.4 mg/dL). ESR was determined by the Westergreen method (Vacuette©ESR, Greiner Bio-One International GmbH, Kremsmünster, Austria). The results were reported in millimeters of fluid (plasma) present in the upper portion of the tube after one hour (mm/h). Normal values vary by gender (range 0–25 for females; 0–20 for males).

### 3.3. Statistical Analysis

Data were analyzed with Student’s *t*-test or the non-parametric unpaired Wilcoxon test for continuous variables. Data are represented as means ± standard deviation (SD). Differences between groups were evaluated using the chi-square test or Fisher’s exact test for categorical variables. Associations showing a *p*-value of less than 0.05 were considered significant.

## 4. Discussion

The present study demonstrated that plasma NGAL levels were higher in patients with psoriasis, as a whole, and in all the subtypes analyzed than those in healthy controls. There are conflicting results regarding the correlation between NGAL and PASI. Consistent with earlier findings [30], we found that plasma NGAL levels did not correlate with PASI, even when evaluated according to different psoriasis subtypes. Thus, NGAL may not completely reflect the degree of skin inflammation of all patients with psoriasis. By contrast, a study by Romani et al. [38] reported a positive correlation between NGAL and PASI. The association between psoriasis and skin cancer is controversial [41,42,43]. Although some authors report that patients with psoriasis have a lower risk of cancer [44], current evidence suggests that they might have a higher risk of NMSC [45]. A recent meta-analysis [46] outlined that psoriatic patients have a 1.72 times higher risk of developing NMSC compared with healthy subjects (relative risk ratio (RR), 1.72, 95% CI 1.46 to 2.02). Moreover, patients with moderate to severe psoriasis had a higher risk of NMSC (RR, 1.82, 95% CI 1.38 to 2.41) than those with mild psoriasis (RR, 1.61, 95% CI 1.25 to 2.09). The risk of developing SCC seems to be significantly higher (RR, 2.08, 95% CI 1.53 to 2.83) than basal cell carcinoma (BCC) (RR, 1.28, 95% CI 0.81 to 2.00). The most plausible hypothesis regarding the pathogenetic mechanisms of the development of skin cancers in psoriatic patients combines chronic low-grade inflammation, risk factors for cancer associated with psoriasis (e.g., smoking, alcohol, obesity), and psoriasis treatments [47]. Furthermore, psoriasis and SCC are skin diseases both characterized by keratinocyte dysregulation with overexpression of NGAL and transcription factor 7-like 1 (Tcf7l1), its regulator, as reported by Xu et al. [48] Their in vitro study on human foreskin keratinocytes (HFK) demonstrated that NGAL expression increased with the Tcf7l1 level in HFKs undergoing differentiation and decreased significantly upon Tcf7l1 depletion. Therefore, Tcf7l1 could contribute to SCC pathogenesis, possibly through upregulating the NGAL pathway. Taken together, these data suggest a possible role of NGAL in the pathogenesis of NMSC. However, none of these studies analyzed plasma NGAL levels in patients with psoriasis and NMSC. To our knowledge, our study is the first to report that NGAL plasma levels are higher in the cohort of patients with psoriasis and NMSC compared to patients with isolated psoriasis. These data were confirmed for NMSCs taken together and singularly for SCC and BCC. We also found higher NGAL levels in patients with skin cancers alone, although lower in comparison with patients with NMSC and psoriasis. A possible mechanism through which NGAL could favor the development of NMSC is iron depletion. In fact, cancer cells exhibit an enhanced dependence on iron relative to their normal counterparts, and dysregulation of iron metabolism can increase cancer risk, promoting tumor growth [49]. Moreover, NGAL forms complexes with both MMP-2 and MMP-9 in cancer cells, which are thought to be involved in regulating cell differentiation [40,50,51]. MMP-9 was demonstrated to infiltrate BCC and SCC by in situ hybridization in the stromal fibroblasts around the neoplasm and was found in the reactive eosinophils infiltrating the dermis [52]. Plasma MMP-9 levels were also found to be higher in NMSC patients than controls, as in oral SCC and other epithelial cancers, and associated with a poor disease prognosis [53]. Higher MMP-9 plasma levels were also present in patients with psoriasis and NMSC than in patients with psoriasis alone. Interestingly, in our study, patients with psoriasis who developed NMSC at follow-up showed increased plasma MMP-9 compared to basal MMP-9 levels. Then, our data suggest a possible role of MMP-9 as a biomarker for new NMSC development in patients with psoriasis. However, few patients have been analyzed; thus, studies on a larger population should be performed.

We found higher serum CRP levels in patients with psoriasis and NMSC than in patients with psoriasis alone. Persistent production of CRP reflects the signaling of the innate immune system, and increased CRP levels have been associated with tumor growth [54]. Other case-control studies reported higher levels of CRP in patients with NMSC compared with controls. However, no data about the correlation between NGAL and CPR levels in patients with psoriasis and NMSC were reported before.

In our study, we found lower plasma NGAL levels in patients with psoriasis and melanoma than in patients with psoriasis alone. Our patients presented with superficial melanoma only (melanoma in situ, or Breslow’s thickness ≤ 0.4 mm) without metastatic disease. This is compatible with the findings by Candido et al. [40] who reported a decrease in NGAL expression in metastatic samples of different neoplasms, including melanoma, suggesting a common inactivation pathway of the NGAL gene during distant tumor dissemination. On the contrary, they did not show significant differences in NGAL mRNA expression between primary melanoma and normal tissue. A substantial downregulation of NGAL was observed only in metastatic melanoma versus primary tumors.

Finally, several studies have addressed the association between vitamin D and skin cancers [55,56,57]. While a recent meta-analysis [57] observed no association between high serum levels of 25(OH)-vitamin D and melanoma risk, high 25(OH)-vitamin D levels have been reported to be associated with increased risk of NMSC, particularly of BCC [56,58]. Moreover, some data suggest an inverse association between vitamin D blood levels and melanoma thickness at diagnosis [59]. Our study did not demonstrate statistically significant differences between serum 25(OH)-vitamin D levels in patients with psoriasis and skin cancers compared with psoriatic patients alone, nor a correlation with NGAL levels.

## 5. Conclusions

In conclusion, NGAL and MMP-9 seem to be promising serum biomarkers of psoriasis and NMSC. Indeed, in our study, patients with psoriasis showed higher plasma NGAL and MMP-9 levels than controls. Patients with psoriasis and NMSC showed statistically significant higher plasma NGAL, MMP-9, and CRP levels compared to patients with psoriasis without skin cancers. Moreover, patients with psoriasis who developed NMSC at follow-up showed increased plasma MMP-9 levels compared to basal MMP-9 levels. On the contrary, patients with psoriasis and melanoma demonstrated lower plasma NGAL levels than psoriatic patients without skin cancers. Thus, NGAL seems to play a role in the pathogenesis of NMSC but not in melanoma in patients with psoriasis, and it may represent a promising biomarker of NMSC in psoriasis. However, further studies in a larger population are needed to confirm these data.

## Figures and Tables

**Table 1:** Demographics and clinical features of patients with psoriasis and healthy controls.

	Patients with Psoriasis (n = 98)	Healthy Controls (n = 20)	Patients with Skin Cancers (n = 15)
**Sex** (N,%)			
Female Male	39 (39.8) 59 (60.2)	12 (60) 8 (40)	9 (60) 6 (40)
**Age**			
mean ± SD (range)	53.8 ± 18.2 (19–93)	56.5 ± 10.6 (37–74)	68.8 ± 17.9 (38–95)
**Family history of psoriasis**			
(N,%)	50 (51.0)	0 (100)	0 (100)
**Family history of NMSC** (N,%)	17 (17.3)	0 (100)	3 (20)
**Family history of melanoma** (N,%)	1 (1.0)	0 (100)	1 (6.7)
**Fitzpatrick’s type (N,%)**			
I II III	1 (2.0) 73 (74.5) 24 (24.5)	0 (0) 16 (80) 4 (20)	1 (6.7) 10 (66.7) 4 (26.6)
**Type of psoriasis** (N,%)			
*Plaque Psoriasis* *Guttate Psoriasis* *Palmo-plantar Psoriasis* *Inverse Psoriasis* *Combination of two or more types*	70 (71.4) 8 (8.2) 5 (5.1) 5 (5.1) 10 (10.2)	-	-
**Disease duration** (years)			
mean ± SD (range)	17.5 ± 11.1 (1–49)	-	-
**PASI score**			
Total Female Male (range)	8.5 8 7.9 (2–17)	-	-
**NMSC** (N,%)			
Total Female Male	15 (15.3) 6 (40) 9 (60)	-	12 (80) 8 (53.3) 4 (26.7)
**Melanoma** (N,%)		-	
Total Female Male	8 (53.3) 3 (37.5) 5 (62.5)		3 (20) 2 (66.7) 1 (33.3)

NMSC = non melanoma skin cancers; PASI = psoriasis area and severity index score; -: none.

**Table 2:** Laboratory data of patients with psoriasis and healthy controls.

	Patients (n = 98)	Controls (n = 20)	*p* Value
**NGAL** (ng/mL)	144.2 ± 41.8	17.1 ± 11.7	<0.0001 *
**MMP-2** (ng/mL)	58.1 ± 44.5	170.9 ± 136.7	<0.0001 *
**MMP-9** (ng/mL)	329.5 ± 156.7	72.4 ± 27.6	<0.0001 *
**CRP** (mg/dL)	0.4 ± 0.4	0.2 ± 0.2	0.02 *
**ESR** (mm/h)	15.2 ± 9.5	8.8 ± 3.0	0.004 *
**25(OH)-vitamin D** (ng/mL)	26.1 ± 7.4	28.6 ± 7.4	0.18

NGAL = neutrophil gelatinase-associated lipocalin; MMP = metalloproteinase; CRP = C-reactive protein; ESR = erythrocyte sedimentation rate; * = stastistically significant.

**Table 3:** Clinical and histological characteristics of NMSC in patients with psoriasis.

	BCC	SCC	BCC + SCC
Total (N,%) Female Male	10 (10.2) 4 (40) 6 (60)	4 (4.1) 2 (50) 2 (50)	1 (1.0) 0 1 (100)
**Anatomical site**			
*Head/neck* *Trunk* *Arms*	3 (30.0) 5 (50) 2 (20)	4 (100) 0 0	1 (100) 0 0
**Histologic type**			
*BCC*			
*Superficial*	**9 (90)**	-	1 (100)
*Nodular*	1 (10)	-	0
*SCC*			
*In situ*		3 (75)	0
*invasive*	-	1 (25)	1 (100)

NMSC = non-melanoma skin cancers; BCC = basal cell carcinoma; SCC = squamous cell carcinoma; -: none.

**Table 4:** Clinical and Histological characteristics of melanoma in patients with psoriasis.

**N (%)**	8 (8.16)
**Anatomical site**	
*Head/neck* *Trunk* *Legs*	1 (12.5) 6 (75) 1 (12.5)
**Histologic type**	
Superficial spreading melanoma	8 (100)
**Breslow thickness**	
In situ <0.75 mm	5 (62.5) 3 (37.5)
**Clark level**	
I III III	5 (62.5) 1 (12.5) 2 (25)
**Mitosis (N/mm^2^)**	
0 ≥1	7 (87.5) 1 (12.5)
**Ulceration**	
Yes No	0 (0) 8 (100)
**Vertical growth phase**	
Yes No	7 (87.5) 1 (12.5)
**Melanic pigment**	
Yes No	3 (37.5) 5 (62.5)
**Lymphohistiocytic peritumor infiltrate**	
Yes No	3 (37.5) 5 (62.5)
**Regression**	
Yes *<75%* *≥75%* No	3 (37.5) 1 (12.5) 2 (25) 5 (62.5)
**Solar elastosis**	
Yes No	2 (25) 6 (75)
**Vascular invasion, neurotropism, microsatellitosis**	
Yes No	0 (0) 8 (100)
**Associated nevus**	
Yes No	1 (12.5) 7 (87.5)

**Table 5:** Patients with psoriasis without skin cancers vs. patients with psoriasis and NMSC.

	Psoriasis without Skin Cancers	Psoriasis and NMSC	*p* Value
**Total (N)** Female (N) Male (N)	75 30 45	15 6 9	
**Age** mean ± SD	49.5 ± 17.6	71.1 ± 12.7	<0.0001 *
**PASI score**	8.0 ± 3.2	9.3 ± 2.7	0.14
**NGAL** (ng/mL)	139.9 ± 39.3	182.3 ± 36.6	<0.001 *
**MMP-2** (ng/mL)	55.3 ± 23.4	61.4 ± 74.8	0.56
**MMP-9** (ng/mL)	342.4 ± 172.2	264.0 ± 84.9	0.09
**CRP** (mg/dL)	0.4 ± 0.4	0.6 ± 0.6	0.04 *
**ESR** (mm/h)	14.7 ± 8.8	15.6 ± 9.2	0.72
**25(OH)-vitamin D** (ng/mL)	26.2 ± 7.3	27.4 ± 8.3	0.56

NGAL = neutrophil gelatinase-associated lipocalin; MMP = metalloproteinase; CRP = C-reactive protein; ESR= erythrocyte sedimentation rate; * = stastistically significant.

**Table 6:** Patients with psoriasis without skin cancers vs. patients with psoriasis and melanoma.

	Psoriasis without Skin Cancers	Psoriasis and Melanoma	*p* Value
**Total (N)** Female (N) Male (N)	75 30 45	8 3 5	
**Age** mean ± SD	49.5 ± 17.6	61.9 ± 9.7	0.05 *
**PASI score**	8.0 ± 3.2	6.0 ± 4.9	0.09
**NGAL** (ng/mL)	139.9 ± 39.3	112.9 ± 26.6	0.06
**MMP-2** (ng/mL)	55.3 ± 23.4	77.9 ± 99.9	0.10
**MMP-9** (ng/mL)	342.5 ± 171.2	331.5 ± 69.4	0.86
**CRP** (mg/dL)	0.4 ± 0.4	0.4 ± 0.4	0.91
**ESR** (mm/h)	14.7 ± 8.8	18.7 ± 12.2	0.24
**25(OH)-vitamin D** (ng/mL)	26.2 ± 7.3	23.5 ± 7.6	0.33

NGAL= neutrophil gelatinase-associated lipocalin; MMP= metalloproteinase; CRP= C-reactive protein; ESR= erythrocyte sedimentation rate; * = stastistically significant.

**Table 7:** Clinical and laboratory characteristics of patients with NMSC at follow-up.

	NMSC Type	Anatomical Site	Timing (Months)	NGAL	MMP-2	MMP-9
**Pt 1**						
t0 t1	BCC BCC	Head/neck Head/neck	16	152.9 98.3	32.9 98.3	288.2 534.6
**Pt 2**						
t0 t1	- SCC	- Head/neck	16	183.5 122.0	32.8 98.0	219.3 577.5
**Pt 3**						
t0 t1 t2 t3	BCC BCC BCC BCC	Head/neck Trunk Trunk Trunk	8 4 8	121.0 245.1 221.0 197.6	71.3 36.6 40.3 40.5	240.7 318.5 336.7 390.6

Pt = patient; t = time; NMSC= non-melanoma skin cancers; BCC = basal cell carcinoma; SCC = squamous cell carcinoma; NGAL= neutrophil gelatinase-associated lipocalin; MMP= metalloproteinase; CRP = C-reactive protein; ESR= erythrocyte sedimentation rate.

## Data Availability

Not available.
